# Assessment of Computed Tomography as a Diagnostic Tool for Upper Respiratory Tract Disorders in Sheep

**DOI:** 10.3390/ani15101445

**Published:** 2025-05-16

**Authors:** Enrique Castells, Pablo Quílez, Aurora Ortín, Sergio Villanueva-Saz, Marta Ruiz de Arcaute, María Climent, Marcelo De las Heras, Héctor Ruiz, Teresa Navarro, Delia Lacasta

**Affiliations:** 1 Centro Clínico Veterinario de Zaragoza, Madre Genoveva Torres Morales, 8, 50006 Zaragoza, Spain; kikevet38@gmail.com; 2 Department of Animal Pathology, Veterinary Faculty, Instituto Agroalimentario de Aragón-IA2 (Universidad de Zaragoza-CITA), University of Zaragoza, Miguel Servet 177, 50013 Zaragoza, Spain; pquilez@unizar.es (P.Q.); aortin@unizar.es (A.O.); svs@unizar.es (S.V.-S.); lasheras@unizar.es (M.D.l.H.); hectorruiz353@gmail.com (H.R.); teresanarr@gmail.com (T.N.); 3 Ruminant Clinical Service, Veterinary Faculty, University of Zaragoza, Miguel Servet 177, 50013 Zaragoza, Spain; mariacli@unizar.es; 4 Department of Anatomy, Embryology, and Animal Genetics, Veterinary Faculty, University of Zaragoza, Miguel Servet 177, 50013 Zaragoza, Spain

**Keywords:** computed tomography, diagnostic imaging, sheep, upper respiratory tract diseases

## Abstract

Upper respiratory tract diseases are a major concern in sheep, causing economic losses and affecting animal welfare. Accurate diagnosis is crucial for effective treatment and prevention. This case-based study assessed the use of computed tomography for diagnosing these diseases in sheep. Thirty-three sheep showing clinical signs of one or more upper respiratory tract diseases underwent computed tomography scans, which were compared to clinical diagnoses and postmortem examinations. Results showed that clinical diagnosis matched the postmortem findings in 26 of 38 cases, while computed tomography diagnosis matched in 36 of 38 diagnosed conditions, demonstrating higher accuracy. Computed tomography provided detailed anatomical reconstructions of the respiratory tract, allowing precise detection of lesions, identification of their origin or content, and clear visualisation of surrounding structures. Despite limitations such as high costs, risks related to general anaesthesia, and radiation exposure, its accuracy makes it a valuable tool for managing these diseases. The creation of extensive databases on common diseases affecting sheep could improve the accessibility and effectiveness of computed tomography in veterinary clinics.

## Simple Summary

Sheep are commonly affected by upper respiratory tract disorders, which can lead to significant health issues and economic losses. Accurate diagnosis is essential for appropriate treatment and disease management. This study presents a clinical case series of 33 sheep with upper respiratory signs examined using computed tomography (CT), with findings compared to clinical and postmortem evaluations. CT enabled detailed visualisation of nasal and sinus structures and improved the identification of lesions such as infections, obstructions, and tumours. Although the technique involves limitations such as cost, the need for anaesthesia, and radiation exposure, it proved to be a valuable diagnostic tool. Establishing CT pattern databases may facilitate earlier recognition of these conditions in clinical settings and support better-informed decisions in sheep health management. This approach may also serve as a model for the diagnostic evaluation of upper respiratory tract disorders in other livestock species.

## 1. Introduction

Various respiratory diseases significantly affect ovine production, resulting in substantial economic losses for lambs and adult ewes. The economic impact of these conditions includes mortality, reduced production, growth retardation, and condemnations at the slaughterhouse [1,2,3]. Respiratory diseases are typically classified into two main categories: those affecting the upper respiratory tract and those affecting the lower respiratory tract. This article will focus on disorders specific to the upper respiratory tract in sheep, involving structures such as the nostrils, nasal cavities, nasal turbinates, paranasal sinuses, pharynx, larynx, and trachea. The differential diagnosis of upper respiratory tract disease (URD) in sheep is broad and complex. In Europe, the most common upper respiratory diseases (URDs) include oestrosis, chronic proliferative rhinitis (CPR), enzootic nasal adenocarcinoma (ENA), and obstructive rhinopathy caused by chronic pithomycotoxicosis (OR). All of these disease have similar clinical signs [4]. Less frequently, conditions such as abscesses, sinusitis, and various types of tumours may also occur.

Oestrosis is a worldwide cavitary myiasis caused by the fly *Oestrus ovis* [5], highly prevalent in Mediterranean areas, such as Spain [6]. New diagnostic techniques, such as PCR, rhinoscopy, and ELISA, are being developed to improve detection [7,8]. CPR is characterised by the proliferation of the nasal mucosa associated with chronic inflammation of the ventral nasal turbinate caused by *Salmonella enterica* subsp. *diarizonae* serovar 61: k:1,5 (7). This proliferation may be visible through the nostrils [9]. Lesions are typically bilateral but can occasionally present unilaterally [10]. ENA is a contagious neoplasm of the glandular cells in the ethmoidal turbinate mucosa, associated with the enzootic nasal tumour virus (ENTV-1). For rapid diagnosis, PCR analysis of a tissue sample or nasal swab is the preferred method. Additionally, histological and immunohistochemical techniques are employed for diagnostic confirmation [11,12]. OR results from sporidesmin, a toxin present in the spores of the fungus *Pseudopithomyces chartarum* [13]. Affected animals exhibit bilateral thickening, primarily at the rostral level of the nasal cavity (the alar portion). Additionally, tracheal disorders, such as tracheal crushing, are considered part of URDs. These are frequently observed in adult sheep in intensive and semi-intensive systems and can affect up to 15% of the flock [14]. Management practices, such as the use of inappropriate feeders or competition for food, are contributing factors to tracheal injuries [14], therefore, it is essential to control elements on the farm that could lead to this condition. Given the overlap in clinical signs among upper respiratory tract disorders (URDs), ancillary diagnostic tools are essential to reach a definitive diagnosis [4]. Nasal swabs may help identify infectious agents [9,11], but imaging techniques are particularly valuable in food-producing animals, where definitive confirmation often relies on postmortem examination [4].

Computed tomography (CT) is a well-established diagnostic imaging technique in human and companion animal medicine. It offers advantages such as three-dimensional reconstructions, airway visualisation using specific filters and algorithms, and enhanced lesion characterisation [15,16]. CT images are displayed in grayscale, with tissue density reflected by attenuation values: bone appears white, air appears black, and soft tissues appear in varying shades of grey [17,18]. In veterinary medicine, CT has improved diagnostic capabilities in small animals and, to a lesser extent, in farm species, mainly due to cost and technical constraints. In sheep, CT has been applied in research contexts, including carcass evaluation [19,20], parasitic diseases such as echinococcosis [21], and respiratory disorders [22,23]. Its ability to visualise bone and soft tissue structures of the nasal and paranasal sinuses makes it particularly suitable for investigating head and upper respiratory tract conditions.

This case-based study aims to describe the CT findings observed in sheep diagnosed with URDs and to evaluate the potential of this technique as a diagnostic tool in clinical practice.

## 2. Materials and Methods

### 2.1. Selection of the Animals and Diagnostic Protocol

This case-based study was conducted between June 2016 and November 2023 and included 33 sheep (32 ewes and 1 ram) presenting clinical signs consistent with upper respiratory tract disorders. The animals included in this study were all adult small ruminants of various breeds and farm origins. All animals originated from farms operating under a semi-intensive production system, which included periods of housing, particularly during critical stages such as parturition and lactation. They were referred to the Ruminant Clinical Service (SCRUM) at the Veterinary Faculty of Zaragoza, Spain, for diagnostic evaluation. All cases came from the faculty’s main catchment area, which includes the regions of Aragón, the Basque Country, Navarre, and Valencia. Upon arrival, each sheep was identified with an individual tag and examined following a standardised clinical protocol, with particular emphasis on the respiratory system. All findings were systematically recorded in individual medical records. To further characterise the lesions, a CT exam was performed in all cases.

### 2.2. CT Scan Protocol

CT scans were performed on all animals using a Brivo CT385 two-slice scanner (GE Healthcare, Chicago, IL, USA) in helical mode. Each sheep underwent a single scan, acquiring two series (soft tissue and bone filters) plus an airway algorithm. Scanning parameters were 80 mA and 120 kV. In selected cases (e.g., suspected enzootic nasal adenocarcinoma, tumours, or obstructive rhinopathy), intravenous contrast (Omnipaque 300 mg iodine/mL; 1.5 mL/kg) was administered, with dosage adjusted to body condition.

Anaesthesia was induced with dexmedetomidine hydrochloride at 0.005 mg/kg IV (Dexdomitor^®^ 0.5 mg/mL; Zoetis España, Madrid, Spain) and buprenorphine hydrochloride at 0.01 mg/kg IV (Vetergesic^®^ 0.3 mg/mL; Ceva Salud Animal, Barcelona, Spain), followed by titrated propofol at an initial dose of 1 mg/kg IV, titrated to effect (Propofol Lipuro^®^ 10 mg/mL; B. Braun Medical S.A., Rubí, Spain). After loss of reflexes, animals were intubated, positioned in sternal recumbency, and anaesthesia was maintained with 2% isoflurane in oxygen (IsoFlo^®^; Zoetis España, Madrid, Spain). Due to the nature of upper airway disease, particular care was taken to prevent airway compromise during anaesthesia.

Scans covered the head and cervical region, from the nostrils to the proximal trachea. Animals were immobilised with non-radiopaque straps and plastic headrests to minimise artefacts. Images were reviewed using RadiAnt DICOM Viewer 4.6.9 (Medixant, Poznań, Poland). Diagnostic reports were produced for each case, detailing lesion location, extent, shape, radiodensity changes, and effects on adjacent structures.

### 2.3. Postmortem Examination

Following the imaging procedures, all animals were humanely euthanised in accordance with ethical guidelines to enable a comprehensive postmortem examination. During necropsy, particular attention was given to the upper respiratory tract and adjacent anatomical structures, which were meticulously examined and documented. When relevant, high-resolution photographs were taken, and representative tissue samples were collected for histopathological, microbiological, and molecular analyses, depending on the suspected aetiology. The final diagnosis was established by integrating gross pathological findings with laboratory results and was subsequently compared with the presumptive diagnosis based on computed tomography (CT) imaging to evaluate the diagnostic accuracy and value of the imaging technique.

### 2.4. Histopathology

The tissue samples for pathological diagnosis were fixed in 10% buffered formalin for at least 48 h before being automatically embedded in paraffin, following standard protocols. Sections measuring 4 μm in thickness were then obtained and stained with Carazzi’s haematoxylin and eosin (H&E) in accordance with routine laboratory procedures.

### 2.5. Molecular and Microbiological Diagnosis

The sheep included in this study were evaluated to detect infections caused by pathogens responsible for upper respiratory tract diseases, such as *Salmonella enterica* subspecies *diarizonae* (CPR) and ENTV-1 (ENA). For this purpose, a molecular analysis, q-PCR (real-time polymerase chain reaction), was used to detect the genetic DNA of these pathogens. In cases of abscesses, microbiological culture was also performed to isolate and identify the causative bacteria. A total of 23 samples were sent for analysis, and tests were carried out in the same private laboratory, EXOPOL (Zaragoza, Spain).

### 2.6. Statistical Analysis

Descriptive statistics were used to summarise the dataset. Diagnostic accuracy was assessed by calculating the proportion of correct diagnoses (true positives) for each method. Sensitivity was defined as the ability to correctly identify positive cases, calculated as the number of true positives divided by the sum of true positives and false negatives. All analyses were performed using SPSS Statistics version 28.0 (IBM Corporation, Armonk, NY, USA).

## 3. Results

### 3.1. Case Distribution

The sheep originated from farms located in Aragón (26/33) and the Basque Country (7/33). The breeds represented were Rasa Aragonesa (24/33; 73%), Latxa (7/33; 21%), Merino (1/33; 3%), and mixed breed (1/33; 3%). The average body condition score of the animals was approximately 1.5 out of 5, with ages ranging from 1 to 8 years.

### 3.2. Clinical Findings

The clinical diagnosis was established based on the clinical signs observed in the animals evaluated, with 33 animals studied. The identified conditions included chronic proliferative rhinitis (CPR, *n* = 14), which was characterised by inspiratory dyspnoea, snoring, and deformities in the nasal, frontal, or maxillary bones. Enzootic nasal adenocarcinoma (ENA, *n* = 5) was characterised by inspiratory dyspnoea, nasal discharge, and deformities in the nasal, ethmoidal, frontal, or maxillary bones. Obstructive rhinopathy (OR, *n* = 7) presented with inspiratory dyspnoea and snoring. Tracheal collapse (*n* = 2) was diagnosed through palpation of the tracheal cartilages, despite the absence of clear clinical signs. A tumour was identified (*n* = 1), which was detected clinically and associated with inspiratory dyspnoea. Finally, four animals (*n* = 4) were classified as “no disease” since they did not exhibit clinical signs consistent with upper respiratory tract disorders (URDs). None of the animals showed clinical signs compatible with oestrosis.

### 3.3. Comparison Between Tomographic and Postmortem Findings

Computed tomography and postmortem examinations were performed in 33 animals, yielding a total of 36 distinct CT diagnoses. The conditions identified included chronic proliferative rhinitis (CPR, *n* = 11), enzootic nasal adenocarcinoma (ENA, *n* = 6), obstructive rhinopathy (OR, *n* = 7), oestrosis (*n* = 2), sinusitis (*n* = 3), abscesses (*n* = 3), tracheal crushing (*n* = 2), nasal deviation (*n* = 1), and squamous cell carcinoma (*n* = 1).

#### 3.3.1. Oestrosis

**Figure 1:**
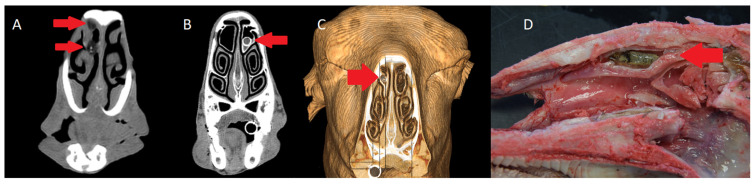
(**A**) Axial view, two larvae of *Oestrus ovis* (red arrows) in left dorsal turbinate (bone filter). (**B**) Axial view; a larva (red arrow) in right dorsal turbinate (bone filter). (**C**) 3D reconstruction of the head of an ewe with a larva (red arrow) in the right turbinate (bone filter). (**D**) Sagittal cross-section of a head with a mummified larva of *Oestrus ovis* (red arrow).

In cases of cavitary myiasis, CT revealed chronic rhinitis with visible *Oestrus ovis* larvae within the nasal cavity. Clear identification was only achieved in the third larval stage (L3), as earlier stages were too small and associated tissue changes were less evident (Figure 1). The macroscopic findings of this pathology include the presence of larvae in different stages and rhinitis associated with their action.

#### 3.3.2. Chronic Proliferative Rhinitis (CPR)

**Figure 2:**
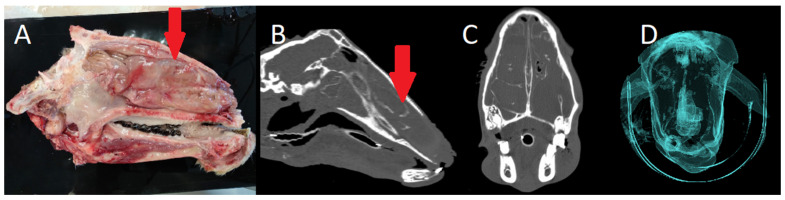
(**A**). Sagittal section of a sheep with severe chronic proliferative rhinitis (CPR) (red arrow). (**B**) CT sagittal view of the same sheep, showing enlargement of the ventral turbinate (red arrow), occupying the entire nasal cavity. No areas of air density are visible, and the normal nasal turbinates are indistinguishable (bone filter). (**C**) CT axial view showing bilateral enlargement of the ventral turbinate, encroaching upon other turbinates and sinuses, with minimal air density observed (bone filter). (**D**) 3D reconstruction of the head, demonstrating the absence of airflow on the left side and significantly reduced airflow on the right side (airway algorithm).

**Figure 3:**
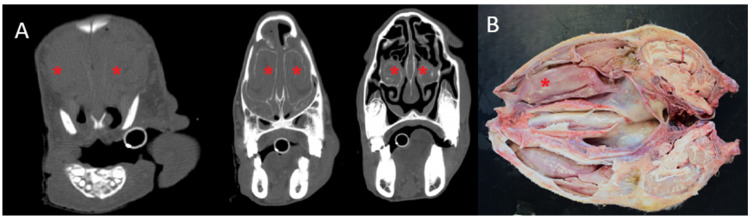
(**A**) Serial axial views of an animal with CPR show bilateral enlargement of the ventral turbinate (red asterisk), with a greater increase in size, resulting in the loss of normal nasal turbinates architecture and nasal septum deformation (bone filter). (**B**) Sagittal section of the same sheep (red asterisk).

CT revealed increased density and volume of the ventral nasal turbinates, with bilateral involvement in nine cases and unilateral involvement in two. The severity ranged from mild mucosal thickening to nasal septum deviation and bone destruction in advanced cases. Similarly, this can be observed as a pathological postmortem finding. CT was particularly valuable for determining lateralisation, lesion extent, and impact on adjacent structures. The airway algorithm (Figure 2 and Figure 3) allowed visualisation of airflow restriction, with decreased passage correlating to the degree of turbinate hypertrophy.

#### 3.3.3. Enzootic Nasal Adenocarcinoma (ENA)

**Figure 4:**
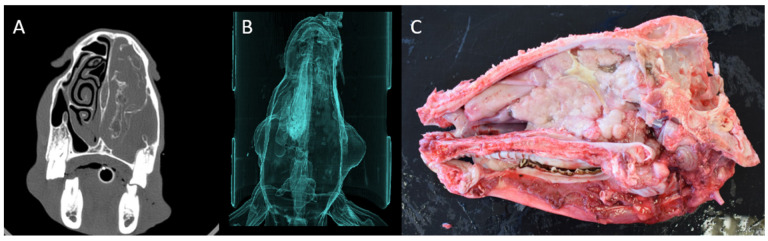
(**A**) Axial view. A unilateral soft tissue density mass occupying the entire right nasal cavity, resulting in the loss of normal architecture of the nasal turbinates and sinuses (bone filter). (**B**) 3D reconstruction of the sheep with ENA shows absent airflow in the right nasal cavity due to tumour obstruction (airway algorithm). (**C**) Sagittal section of the same sheep with ENA. Unilateral tumour mass in the ethmoid turbinate.

Masses arising from the ethmoidal turbinates were observed, with three bilateral and two unilateral cases (one case was inconclusive due to advanced destruction). The tumours disrupted the ethmoidal structures and frequently exhibited polypoid extensions. In advanced cases, CT revealed destruction of the ethmoid and nasal bones, involvement of paranasal sinuses, and exophthalmos due to soft tissue expansion. Post-contrast imaging highlighted increased tissue density in the ethmoidal region. The airway algorithm demonstrated partial or complete replacement of air-filled spaces by tumour masses (Figure 4). These same findings were observed in the postmortem study, with the help of a sagittal section of the head to examine the tumour.

#### 3.3.4. Obstructive Rhinopathy (OR)

**Figure 5:**
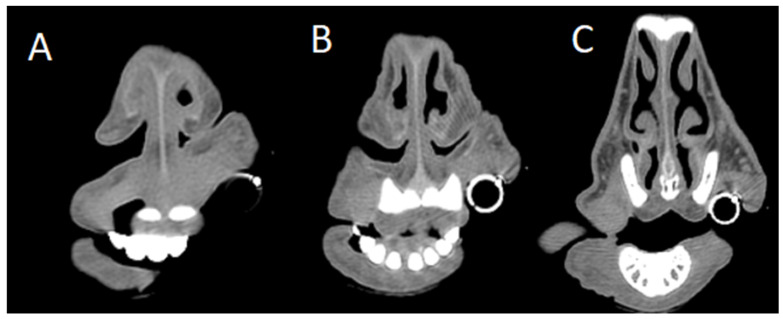
Serial axial computed tomography (CT) sections of a sheep affected by obstructive rhinopathy due to chronic pithomycotoxicosis (bone filter). (**A**) Most rostral section showing a soft tissue proliferation within the anterior nasal cavity, resulting in partial obstruction of the nasal passages. (**B**) Mid-level section demonstrating a reduced extent of soft tissue proliferation and a lower degree of obstruction. (**C**) Caudal section with no detectable soft tissue proliferation or nasal cavity obstruction.

**Figure 6:**
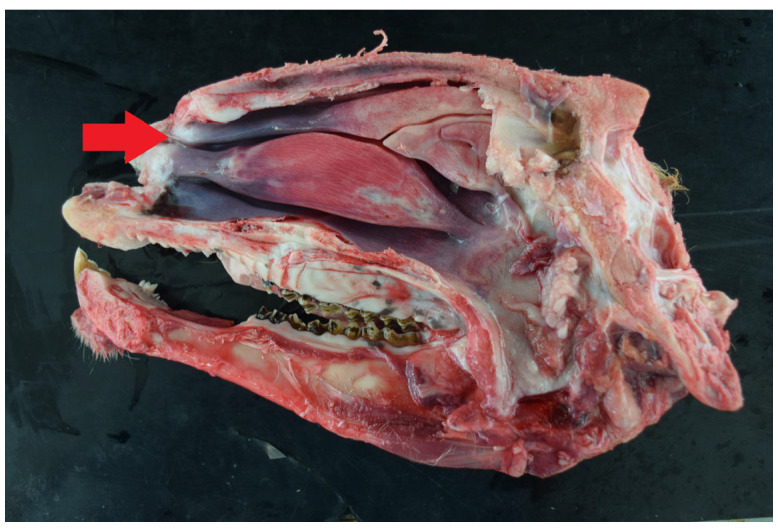
In the sagittal section of an affected sheep with obstructive rhinopathy; thickening is observed in the alar portion of the ventral turbinate (red arrow).

CT findings included soft tissue proliferation in the rostral nasal cavity with variable degrees of obstruction. In serial cross-sections, lesions were confined to the anterior portion, with decreasing soft tissue density in caudal regions (Figure 5). All cases showed bilateral involvement, although asymmetrical distribution was occasionally observed (Figure 6). Intravenous contrast administration did not significantly improve lesion differentiation. As postmortem findings, nasal canal narrowing can be visualised in a frontal view of the nostrils, clearly shown with a sagittal section of the skull, where the small, affected area is visible.

#### 3.3.5. Squamous Cell Carcinoma

**Figure 7:**
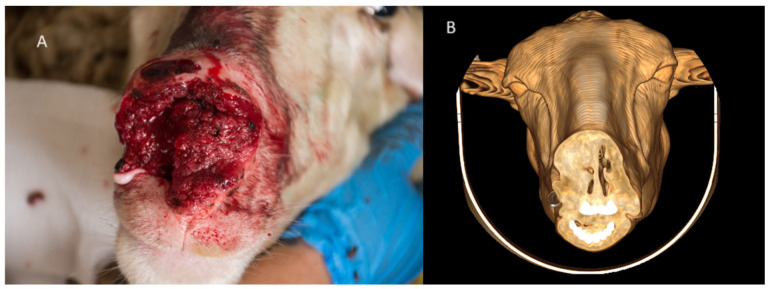
(**A**). Sheep with proliferative, ulcerative, and bleeding lesions in the nostrils. (**B**) 3D reconstruction of the head with an axial cut at the level of the nostrils, showing proliferation and tissue infiltration on the right side, obstructing the nasal meatus.

As shown in the CT images (Figure 7), the tumour exhibited marked infiltration and tissue destruction, with significant alteration of the normal anatomy of the nostrils and nasal passages. Tumour growth was more pronounced on the right side, resulting in complete obstruction of the right nasal passage. Airflow analysis revealed bilateral impairment, with total absence of airflow through the right nasal passage, which correlated with the observed inspiratory dyspnoea. Given the neoplastic nature of the lesion, a full-body CT scan was performed to assess for potential metastases. The lesion was clearly visible in the postmortem macroscopic examination. No evidence of metastasis was found in any internal organ.

#### 3.3.6. Nasal Septum Deviation

**Figure 8:**
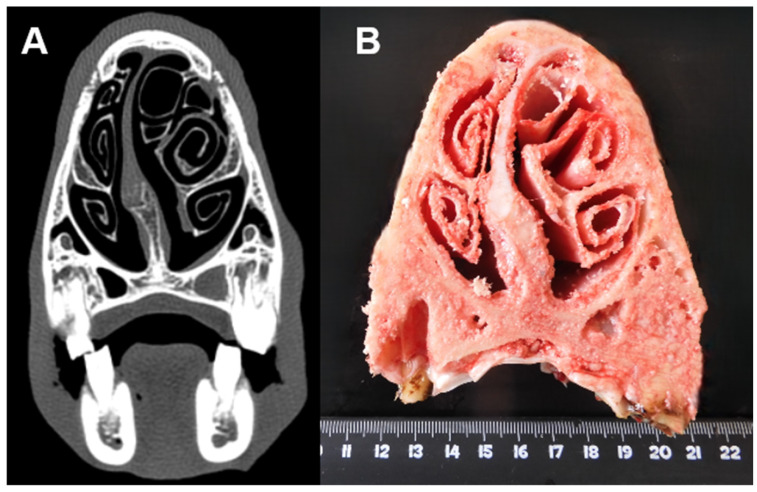
(**A**) Axial view; deviation of the nasal septum without any other evident disease. (**B**) Axial section of the same head.

In the CT scan, axial and coronal sections revealed a deviation of the nasal septum to the left, resulting in a reduction in the size of the dorsal and ventral nasal turbinates on the right side compared to those on the left side. Additionally, the thickening of the medial part of the left ventral turbinate and the ethmoid bone was also evident (Figure 8). In the postmortem study, nasal deviation cannot be observed without cutting the animal’s skull in order to examine the nasal septum.

#### 3.3.7. Tracheal Crushing

**Figure 9:**
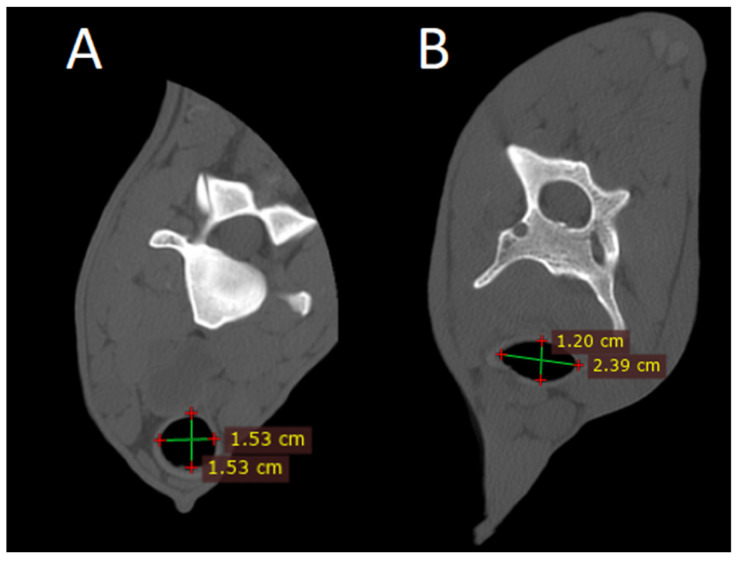
(**A**) Axial view of the neck with a normal trachea (bone filter). (**B**) Axial view of the neck with tracheal crushing (bone filter).

An unusual finding of tracheal collapse was found in a CT review. In a normal axial CT image, the tracheal lumen is expected to maintain a uniform, rounded diameter (Figure 9). Deviations from this symmetry suggest the presence of collapse. In the postmortem study, this narrowing is observed in the ventrodorsal diameter in comparison to the laterolateral diameter.

#### 3.3.8. Sinusitis

**Figure 10:**
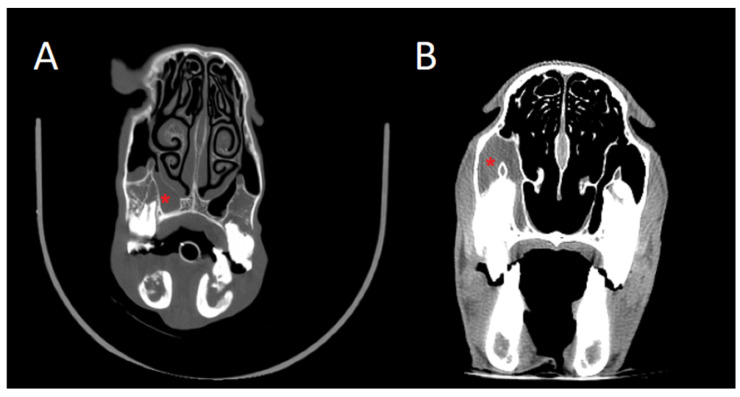
(**A**) Axial view of a sheep’s skull with left maxillary sinusitis, showing complete accumulation of isodense material in the sinus (red asterisk). (**B**) Axial view of another sheep with ventral accumulation of isodense material in the left maxillary sinus, causing sinusitis (red asterisk).

In the CT findings of sinusitis, accumulation of purulent material was observed within the sinuses, most commonly in the maxillary sinuses. In some cases, this led to deformation of the ventral turbinates and facial contour, with potential involvement of adjacent bone structures and the formation of fistulas (Figure 10). The extension of nasal abscesses into neighbouring cavities could also be detected. These macroscopic findings, including the purulent content, were confirmed postmortem following euthanasia and cranial dissection of the affected animals.

#### 3.3.9. Abscesses

**Figure 11:**
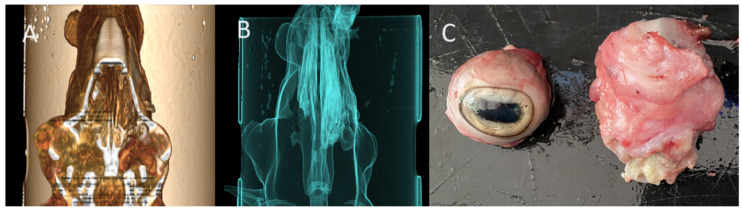
(**A**) 3D reconstruction of the head; axial cut showing a retrobulbar mass protruding from the left eye. (**B**) Same head; airflow algorithm demonstrating no airflow passage from the left ethmoidal labyrinth. (**C**) Retrobulbar abscess on postmortem examination.

In one ewe, CT revealed a mass occupying the ethmoidal sinus, extending towards the dorsal concha, auricular pavilion, and left cheek, with the presence of a retrobulbar abscess. Additionally, radiolucent areas compatible with fluid accumulation or low-consistency material were observed, suggesting a differential diagnosis of a retrobulbar tumour. The postmortem examination confirmed the presence of a retrobulbar abscess protruding outward from the eye (Figure 11).

**Figure 12:**
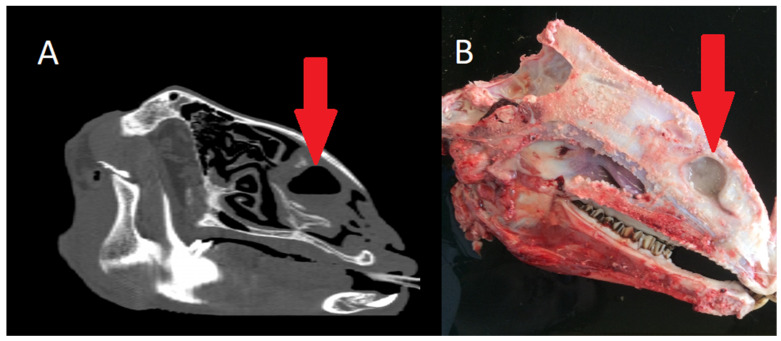
(**A**) Sagittal view of a sheep’s head with a rounded structure in the nasal septum invading the nasal turbinates and with low-density content consistent with an abscess (red arrow) (bone filter). (**B**) Sagittal section of the same sheep with a nasal abscess (red arrow) containing purulent content in the nasal septum.

In another two animals, nasal abscesses were detected. CT imaging provides a comprehensive assessment of the abscess (Figure 12), allowing precise determination of its size and location within the nasal cavity and differentiation of its contents. Additionally, CT enables the identification of damaged tissues and affected areas and the detection of bone lesions, including the thinning of the bone walls due to abscess progression. In certain cases, it may also reveal the presence of gas within the abscess or adjacent tissue destruction, helping to guide appropriate treatment strategies.

### 3.4. Histopathological Findings

To confirm the macroscopic findings, twenty-nine samples were processed for histopathological examination. The results confirmed the diagnoses of oestrosis (*n* = 4), chronic proliferative rhinitis (CPR; *n* = 11), enzootic nasal adenocarcinoma (ENA; *n* = 6), obstructive respiratory pathology (ORP; *n* = 7), and squamous cell carcinoma (*n* = 1).

#### 3.4.1. Oestrosis

Lesions found ranged from moderate to severe rhinitis, with focal to extensive, proliferative, and granulomatous patterns. Multifocal ulceration was noted at larval attachment sites. Goblet cell, mucous cell, and glandular hyperplasia were commonly observed. Chronic cases showed submucosal fibrosis and marked neovascularisation of the affected tissue.

#### 3.4.2. Chronic Proliferative Rhinitis (CPR)

Histological sections revealed a thickening of the nasal mucosa in the ventral turbinates, with polypoid projections and epithelial hyperplasia containing intracellular Gram-negative bacilli. The lamina propria exhibited neutrophilic infiltration, eosinophilic material, and expansion of plasma cells.

#### 3.4.3. Enzootic Nasal Adenocarcinoma (ENA)

Proliferation of secretory epithelial cells was observed in the ethmoidal turbinates. The stroma was infiltrated by lymphocytes and plasma cells, either scattered or clustered. Neoplastic cells corresponded to serous, mucous, or mixed-type glandular epithelium.

#### 3.4.4. Obstructive Rhinopathy (OR)

Lesions were characterised by arteriosclerosis, with degenerative changes in the small arteries of the alar folds and surrounding tissue.

#### 3.4.5. Squamous Cell Carcinoma

Histopathology showed well-demarcated neoplastic foci of epithelial cells without basement membrane invasion. Orthokeratotic hyperkeratosis and dysplasia were present in areas with increased keratin accumulation. Mild hyperplasia of the spinous and basal layers was observed, along with superficial dermal infiltration by lymphocytes, macrophages, and occasional neutrophils. Necrosis of individual keratinocytes and serocellular crusts containing parakeratotic keratin and neutrophils were also noted.

### 3.5. Molecular and Microbiological Findings

All 11 animals clinically suspected of CPR tested positive by RT-PCR for *Salmonella enterica* subspecies *diarizonae*, the causative agent of this condition. All six animals suspected of having ENA tested positive by RT-PCR for ENTV-1, confirming infection of the ethmoidal turbinate. In the six cases of abscesses and sinusitis, microbiological culture identified *Pasteurella multocida*, *Escherichia coli*, and *Corynebacterium pseudotuberculosis* as the primary isolated pathogens.

### 3.6. Diagnostic Evaluation

A total of 38 upper respiratory tract disorders were diagnosed in 33 animals, with some individuals presenting more than one condition. The distribution of pathological diagnoses as final diagnosis was as follows: CPR in 11 cases (11/38:28.9%), OR in 7 (7/38: 18.4%), ENA in 6 (6/38: 15.8%), oestrosis in 4 (4/38: 10.5%), sinusitis in 3 (3/38: 7.9%), upper respiratory tract abscesses in 3 (3/38: 7.9%), tracheal crushing in 2 (2/38: 5.3%), and 1 case each of nasal deviation and other neoplasia (1/38: 2.6% each) (Table 1).

The study compared the diagnoses obtained through clinical examination and computed tomography (CT) with the pathological diagnosis, considered the gold standard. CT demonstrated greater diagnostic accuracy (94.7%) compared to clinical examination (68.4%), highlighting its value as a reliable diagnostic tool. While clinical examination provides important initial insights, CT significantly improves diagnostic precision by detecting a higher number of cases and showing closer agreement with postmortem findings. The discrepancies observed in certain cases underscore the importance of using complementary diagnostic methods to achieve a more complete and accurate veterinary assessment.

## 4. Discussion

Previous studies have highlighted the challenges of diagnosing URDs in sheep, given their diverse aetiologies and overlapping clinical signs [24,25]. URDs in sheep encompass a wide range of conditions, including infectious agents, parasites, allergic reactions, and neoplasms [24]. Despite their varied origins, these diseases often present with similar clinical signs, which can make differential diagnosis challenging. Although the overall incidence of URDs in sheep is generally considered low compared to lower respiratory tract diseases [25], certain conditions, such as oestrosis and OR, can affect entire flocks, with significant impacts when outbreaks occur [6,13]. ENA has also been reported in outbreaks with a prevalence exceeding 16% [26]. However, our study confirms that CT can overcome many of these challenges by providing detailed anatomical reconstructions that allow precise localisation of lesions and their relationship with surrounding tissues.

The disease distribution described in this study should not be interpreted as representative of the general sheep population. As a referral centre, the SCRUM service receives a higher proportion of complex or unresolved cases. In addition, the research team has a specific focus on upper respiratory tract pathologies, which may contribute to selection bias. In three animals, L3 larvae of *Oestrus ovis* were found in conjunction with other diseases. Oestrosis was not considered the primary cause of clinical signs in these cases. CT was unable to detect the parasite in two animals, likely due to the presence of larvae in early stages, which are too small to visualise and cause minimal inflammation [22]. Detection was only possible in the L3 stage. Moreover, it is important to mention that the causative agent of CPR, *Salmonella enterica* subspecies *diarizonae*, is commonly present in the nasal turbinates of asymptomatic sheep [27], which is why histopathological confirmation is always required to support the diagnosis.

Given their potential clinical and economic impact, these diseases require accurate and timely diagnosis to enable effective treatment and control strategies. Imaging techniques are key in achieving this goal. CT offers several advantages over other diagnostic imaging modalities, such as thermography and radiography. Although ultrasound is widely used for diagnosing various conditions in sheep, its application in URDs is limited due to the presence of air-filled cavities and dense cranial bone, which hinder image acquisition. Nevertheless, ultrasound may be useful for evaluating certain lesions located in the neck region.

Among the imaging techniques available for URDs in sheep, thermography and radiography are more practical for field use. Thermography is quick, non-invasive, and does not require anaesthesia. It detects inflammation by capturing variations in surface temperature [28] and has shown utility in detecting sinusitis, ENA, and CPR [22,29], although its effectiveness is limited in abscesses and absent in oestrosis. Our experience also suggests limited utility in obstructive rhinopathy. Radiography, commonly used in small animal and equine practice [30], is less suitable for sheep due to the anatomical complexity of the nasal region and the limitations of two-dimensional imaging, which can result in overlapping structures and reduced interpretability [18,22]. Another technique available for the evaluation of URDs is endoscopy, which has proven to be a simple, swift, and safe procedure in small ruminants, allowing direct visualisation of mucosal surfaces and anatomical structures, as well as the collection of biopsies [31]. While it constitutes a highly useful supplementary diagnostic tool, its scope may be limited in detecting deep lesions or in anatomically complex areas.

The comparison between clinical and CT-based diagnoses in this study highlights the superior diagnostic performance of computed tomography. While clinical evaluation is essential for initial assessment, it may fail to identify deep-seated or symmetrical lesions. In this case series, CT findings were consistent with postmortem diagnosis in 31 of 33 cases, compared to 26 of 33 for clinical evaluation. This difference was particularly relevant in animals with multiple or complex conditions, where CT allowed detailed visualisation of lesion location, extent, and relationship with surrounding tissues. However, the results showed that CT diagnosis achieved a higher diagnostic accuracy (36 out of 38 diagnosed conditions) compared to clinical examination (26 out of 38 diagnosed conditions), reinforcing its value as a more precise and reliable diagnostic tool for upper respiratory tract disorders in sheep.

CT enables detailed anatomical reconstructions of the upper respiratory tract and facilitates accurate lesion characterisation. The use of post-contrast imaging, multiplanar reconstructions, and airway algorithms enhances the evaluation of both soft tissue and air-filled structures, allowing identification of obstructions and associated anatomical changes. Nevertheless, CT has several limitations, including high cost, the need for general anaesthesia, and higher radiation exposure compared to other imaging modalities [32]. These factors currently limit its use in field conditions. However, CT is routinely used in small animal clinics for respiratory assessment [33,34], and its application in sheep may expand with continued technological improvements.

## 5. Conclusions

Computed tomography proved to be a highly effective tool for the diagnosis of upper respiratory tract disorders in sheep, with superior diagnostic accuracy compared to clinical examination. Its ability to visualise complex anatomical structures, differentiate overlapping tissues, and assess airflow makes it especially valuable in cases involving multiple or ambiguous lesions. Although its routine use in livestock is currently limited by technical and economic constraints, CT holds strong potential for specialised veterinary practice. Establishing reference imaging databases for common respiratory diseases in sheep will be essential to support broader clinical application and to enhance early, accurate diagnosis in both production and companion animals.

## Figures and Tables

**Table 1:** Comparison of the clinical, tomographic and pathological diagnosis (gold standard) of the studied diseases of sheep with different diseases.

Disease	Pathological Diagnosis	Clinical Diagnosis	CT Diagnosis	True Positive Clinical Diagnosis	True Positive CT Diagnosis
CPR	11	14	11	11	11
ENA	6	5	6	5	6
Oestrosis	4	0	2	0	2
OR	7	7	7	7	7
Tracheal crushing	2	2	2	2	2
Sinusitis	3	0	3	0	3
Abscess	3	0	3	0	3
Nasal deviation	1	0	1	0	1
Tumour	1	1	1	1	1
No disease	0	4	0	0	0
Total Diagnosis	38	33	36	26	36

Abbreviations: CPR, chronic proliferative rhinitis; ENA, enzootic nasal adenocarcinoma; OR, obstructive rhinopathy.

## Data Availability

The datasets generated during and/or analysed during the current study are available from the corresponding author on reasonable request.
